# Predictive Modeling of Osteonecrosis of the Femoral Head Progression Using MobileNetV3_Large and Long Short-Term Memory Network: Novel Approach

**DOI:** 10.2196/66727

**Published:** 2025-08-06

**Authors:** Gang Kong, Qi Zhang, Dan Liu, Jingbo Pan, Kegui Liu

**Affiliations:** 1 Yantaishan Hospital, No.91 Jiefang Road, Zhifu District, Yantai, 264000, China, 86 13395358569

**Keywords:** artificial intelligence, deep learning, disease progression, MobileNetV3_Large, osteonecrosis of the femoral head, pathological image analysis, treatment response evaluation, vascular reconstruction

## Abstract

**Background:**

The assessment of osteonecrosis of the femoral head (ONFH) often presents challenges in accuracy and efficiency. Traditional methods rely on imaging studies and clinical judgment, prompting the need for advanced approaches. This study aims to use deep learning algorithms to enhance disease assessment and prediction in ONFH, optimizing treatment strategies.

**Objective:**

The primary objective of this research is to analyze pathological images of ONFH using advanced deep learning algorithms to evaluate treatment response, vascular reconstruction, and disease progression. By identifying the most effective algorithm, this study seeks to equip clinicians with precise tools for disease assessment and prediction.

**Methods:**

Magnetic resonance imaging (MRI) data from 30 patients diagnosed with ONFH were collected, totaling 1200 slices, which included 675 slices with lesions and 225 normal slices. The dataset was divided into training (630 slices), validation (135 slices), and test (135 slices) sets. A total of 10 deep learning algorithms were tested for training and optimization, and MobileNetV3_Large was identified as the optimal model for subsequent analyses. This model was applied for quantifying vascular reconstruction, evaluating treatment responses, and assessing lesion progression. In addition, a long short-term memory (LSTM) model was integrated for the dynamic prediction of time-series data.

**Results:**

The MobileNetV3_Large model demonstrated an accuracy of 96.5% (95% CI 95.1%‐97.8%) and a recall of 94.8% (95% CI 93.2%‐96.4%) in ONFH diagnosis, significantly outperforming DenseNet201 (87.3%; *P*<.05). Quantitative evaluation of treatment responses showed that vascularized bone grafting resulted in an average increase of 12.4 mm in vascular length (95% CI 11.2‐13.6 mm; *P*<.01) and an increase of 2.7 in branch count (95% CI 2.3‐3.1; *P*<.01) among the 30 patients. The model achieved an AUC of 0.92 (95% CI 0.90‐0.94) for predicting lesion progression, outperforming traditional methods like ResNet50 (AUC=0.85; *P*<.01). Predictions were consistent with clinical observations in 92.5% of cases (24/26).

**Conclusions:**

The application of deep learning algorithms in examining treatment response, vascular reconstruction, and disease progression in ONFH presents notable advantages. This study offers clinicians a precise tool for disease assessment and highlights the significance of using advanced technological solutions in health care practice.

## Introduction

Osteonecrosis of the femoral head (ONFH) is a rapidly progressive and impactful orthopedic disease with complex etiology, involving both traumatic factors (eg, hip injuries) and nontraumatic factors (eg, prolonged corticosteroid use, alcohol abuse, and metabolic disorders) [1,2]. These causative factors collectively lead to local interruption of blood supply, triggering osteocyte necrosis, which can ultimately result in trabecular bone collapse and irreversible loss of joint function. ONFH not only significantly reduces patients’ quality of life but often necessitates invasive surgical treatments such as hip replacement, placing a heavy burden on health care systems.

Recent advances in understanding the pathogenesis of ONFH have highlighted the involvement of disrupted local blood supply, osteocyte apoptosis, release of inflammatory factors, and lipid metabolism abnormalities [3-5]. Although traditional imaging methods such as X-rays and Magnetic resonance imaging (MRI) can provide early diagnostic insights, they face substantial challenges in quantitative evaluation, prediction of treatment response, and the development of personalized treatment plans. For example, X-rays have low sensitivity for detecting early lesions, often delaying diagnosis [6], while MRI, despite its high soft tissue resolution, lacks quantitative analysis capabilities for posttreatment changes in lesions [7,8]. Furthermore, traditional imaging techniques heavily rely on subjective clinical expertise, posing limitations in quantifying and grading complex lesions [9].

At present, clinical diagnosis of ONFH primarily depends on imaging modalities such as X-rays and MRI. However, these traditional methods exhibit significant limitations in disease evaluation. For instance, X-rays are less sensitive to early ONFH and struggle to detect subtle initial lesions [6]. Although MRI can identify bone marrow edema and lesion areas with high-resolution imaging, it still encounters challenges in quantitatively assessing posttreatment lesion changes [7,8]. Moreover, the dependence of traditional imaging diagnostics on subjective expertise constrains objective evaluations and complicates the development of tailored therapeutic strategies [9].

With the rapid advancements in artificial intelligence (AI), particularly deep learning technologies, medical imaging analysis has entered a new era. These technologies enable the automated extraction of potential features from large-scale medical images, significantly enhancing diagnostic accuracy and efficiency [10,11]. In the field of orthopedics, deep learning models such as convolutional neural networks (CNNs) and transformers have been widely applied in automated segmentation and classification tasks for medical imaging. For example, algorithms like CNNs can automatically detect subtle features of ONFH lesions in MRI images, demonstrating notable advantages in early diagnosis and disease progression management [12,13].

Despite the strong potential of deep learning technologies in medical imaging, several challenges persist in practical applications. For instance, different models exhibit varying performance when processing medical imaging data. Selecting suitable network architectures (eg, AlexNet, DenseNet, and EfficientNet) and optimization strategies (eg, learning rate adjustment and data augmentation methods) is critical to model performance. For a complex condition like ONFH, deep learning models must achieve high sensitivity and specificity to accurately detect early lesions and provide reliable predictions. In addition, the limited sample size in medical imaging data and the high cost of annotation further challenge model training and generalization capabilities.

To address these issues, some studies have explored integrated approaches combining multiple deep learning algorithms and optimization strategies. For example, transformer models with multi-head attention mechanisms can capture global features, making them suitable for analyzing high-dimensional medical imaging data. Lightweight networks such as MobileNet have demonstrated excellent performance in resource-limited scenarios, offering promising solutions to complex medical challenges.

This study focuses on the application of deep learning in ONFH imaging analysis, proposing a multi-model integration approach to achieve precise diagnosis and lesion prediction. The research showcases several innovative aspects. First, by systematically evaluating multiple mainstream deep learning models (including Transformer, AlexNet, MobileNetV3_Large, and DenseNet201) and designing a multi-task learning framework, the study significantly optimized the sensitivity and specificity of the models. Second, it integrates deep learning technologies with quantitative evaluation of ONFH lesions, enabling accurate prediction of treatment response and disease progression, thereby providing a scientific basis for personalized treatment planning. Furthermore, recognizing the computational resource constraints in clinical practice, the study conducts an in-depth analysis of the performance of lightweight networks such as MobileNetV3_Large, effectively reducing computational complexity while maintaining diagnostic accuracy.

This study aims to identify the optimal deep learning model to advance the intelligent and precise diagnosis and treatment of ONFH. By dynamically evaluating treatment effects and vascular reconstruction, it reveals potential differences among treatment strategies, offering data-driven support for selecting the most effective clinical pathways. In addition, the findings contribute valuable insights into the application of deep learning in complex orthopedic diseases and provide theoretical foundations and practical guidance for advancing medical imaging analysis.

## Methods

### Clinical Sample Collection

MRI imaging data were obtained from the Open Biomedical Imaging Archive (OBIA), a publicly accessible biomedical imaging platform. All data were deidentified in accordance with strict protocols. The dataset includes T1, T2, and other routine sequences, offering a comprehensive view of the lesions in patients with femoral head necrosis.

Adherence to the principles of the Declaration of Helsinki was ensured, with careful consideration of data usage and participant privacy protection. Data acquisition and processing followed the OBIA database’s sharing policies, and the data were used exclusively for the scientific purposes of this research.

### Image Preprocessing

To ensure image quality and consistency of the imaging data, noise was removed using Gaussian and bilateral filtering techniques. Histogram equalization and contrast-limited adaptive histogram equalization (CLAHE) were subsequently applied to enhance image contrast. All images were then resized to a uniform dimension of 256×256 pixels to ensure consistency in the input data. These preprocessing steps were implemented using ImageJ and custom Python scripts.

### Data Annotation

Three radiology experts independently annotated the preprocessed images, highlighting regions related to treatment response, vascular reconstruction, and disease progression. The multi-label annotation was conducted using the LabelMe tool (MIT). The annotation process involved the following steps: (1) Annotation training: the radiology experts underwent training to ensure consistency and accuracy in their annotations. (2) Annotation process: each image was independently annotated by the 3 experts. The annotations were then reviewed by 5 orthopedic specialists to establish the ground truth. Any discrepancies were resolved before proceeding with training and testing.

### Dataset Division

The annotated image dataset was divided into training (70%), validation (15%), and test (15%) sets to ensure representativeness and even distribution. Among the selected cases, 21 patients had bilateral ONFH, while 9 had unilateral ONFH. The unaffected hips of these 9 patients were used as a control group. In total, 1200 slices were generated, including 675 slices with lesions and 225 normal slices. Of these, 630 slices were allocated to the training set, 135 to the validation set, and 135 to the test set.

### Algorithm Model Selection

In this study, we selected ten deep learning algorithms for initial model training, including Transformer, AlexNet, MobileNetV3_Large, InceptionV4, InceptionResNetV2, EfficientNetB0, DenseNet201, DarkNet_Small, VGG16, and SEResNet50 (Figure S1 in Multimedia Appendix 1). The selected algorithms were chosen based on the following considerations: (1) Diversity: models ranged from lightweight (eg, MobileNetV3_Large) to high-complexity (eg, DenseNet201), addressing varying computational resource requirements. (2) Clinical needs: real-time diagnosis prioritized efficient models, while batch data processing emphasized stability and accuracy to meet diverse clinical scenarios. (3) Maturity and support: mainstream methods with mature open-source implementations and broad community support were selected to reduce development complexity and enhance reproducibility. (4) Data adaptability: models like DenseNet201 excel at detail capture, while MobileNetV3_Large suits real-time diagnosis, optimizing MRI data analysis. (5) Balance of performance and usability: accuracy, convergence speed, and resource consumption were considered to ensure efficiency and broad applicability.

### Model Training

Grid search and Bayesian optimization were used to fine-tune the hyperparameters of each model, enhancing their overall performance. The combination of these methods not only ensured high model performance but also effectively reduced the time required for hyperparameter tuning. To evaluate model performance, cross-validation was used. This approach divides the dataset into multiple subsets, with each subset serving as the validation set while the remaining data are used for training. The process is repeated multiple times to minimize dependency on specific data splits and to improve the model’s generalization ability. The key evaluation metrics included accuracy, precision, recall, and *F*_1_-score, which collectively provide a comprehensive assessment of the model’s performance in classification tasks. Accuracy indicates the overall correctness of the model’s classifications, precision measures the accuracy of positive predictions, recall reflects the model’s ability to capture all relevant positive instances, and the *F*_1_-score, being the harmonic mean of precision and recall, offers a balanced evaluation of the model’s classification effectiveness.

### Feature Extraction

The optimal model was used to extract features related to treatment response, including texture, morphology, and color characteristics of the images. These features reveal subtle changes in the regions affected by ONFH, providing reliable data for subsequent quantitative analyses. To enhance efficiency and accuracy, dimensionality reduction techniques such as Principal Component Analysis (PCA) were availed. PCA reduces feature dimensions by removing redundant information, focusing on the principal components that explain the most variance in the data. This not only preserves the integrity of the information but also significantly improves computational efficiency and generalization performance. Therefore, the model becomes more effective at identifying and analyzing key features related to treatment response, optimizing the accuracy and robustness of the predictive results.

### Evaluation of Treatment Efficacy

Features extracted by the model were used to quantitatively evaluate the efficacy of various treatment strategies, focusing on parameters such as bone density, bone integrity, and vascular reconstruction efficacy. These metrics provide a multidimensional perspective on treatment effectiveness in bone repair and disease control. Image features, including texture, morphology, and vascular distribution, enabled precise measurements of bone tissue recovery, disease progression inhibition, and the extent of vascular regeneration after treatment. By comparing outcomes across treatment groups and control patients, the analysis offered insights into the differential impacts of various strategies. Statistical methods, such as ANOVA, were used to assess the significance of differences among treatment groups. ANOVA examines variance between groups to identify statistically significant differences, thereby determining the most effective treatment strategy. This approach provides a clearer understanding of how different strategies contribute to the improvement of ONFH.

### Image Segmentation Using the U-Net Model

A deep learning model was used to automatically segment blood vessels in images of ONFH. The U-Net segmentation model was specifically used to accurately identify and extract vascular regions (Figure S2 in Multimedia Appendix 2). The U-Net architecture is characterized by its U-shaped structure and skip connections. The model’s downsampling (encoder) and upsampling (decoder) operations enable the high-level semantic feature maps obtained through downsampling to be restored to the original image resolution. Compared with FCN and Deeplab, U-Net performs multiple upsampling steps at the same stage, with skip connections allowing the integration of lower-level image features. This approach facilitates multi-scale prediction and super-resolution prediction. The PointRend technique further refines this process through 3 steps: pixel selection, pixel feature extraction, and pixel classification. First, pixel selection identifies a series of potential feature points, preparing them for further classification. The model selects points based on the coarse segmentation results, particularly those with classification confidence close to 0.5 (indicating uncertainty in classification). These points are typically located near the edges of objects. Next, feature extraction is performed on the selected points, the coarse segmentation network extracts the features at the corresponding locations. Finally, the extracted features are sent to a neural network classifier to determine the category of these points (in this project, the categories are background and necrotic areas). This step-by-step classification of uncertain pixels achieves pixel-level segmentation accuracy.

### Vascular Feature Extraction and Evaluation

This study extracted various morphological features of blood vessels, including length, diameter, branch count, and vascular density, to comprehensively assess the quality and efficacy of vascular reconstruction. Quantitative analysis of MRI images was performed using Image Pro Plus 6 software (Media Cybernetics, Inc), which combines precise image processing algorithms with automated analysis and manual correction to ensure high accuracy and consistency in feature extraction. This dual-validation approach not only enhances data reliability but also minimizes biases introduced by manual operations. After feature extraction, statistical methods such as *t* tests were used to analyze these vascular characteristics in detail, comparing the impact of different treatment methods on vascular morphology. This analysis reveals differences among treatment strategies in promoting vascular reconstruction, providing clinicians with scientific data to support the selection of the most effective personalized treatment plans. Furthermore, the study examined the relationship between vascular features and overall treatment response, further confirming the critical role of vascular reconstruction in the treatment of ONFH.

### Time-Series Data Preparation and Progression Prediction

Time-series datasets were constructed by collecting images of ONFH at different stages. Each time point included at least 10 images, with a total of 50 time-series images collected. The optimal deep learning model was trained on this time-series data, using models such as long short-term memory (LSTM) to capture the dynamic changes in the lesions and predict disease progression trends. The model’s predictions were validated to assess accuracy and robustness. Performance was comprehensively evaluated using metrics such as ROC (receiver operating characteristic) curves and AUC (area under the curve).

### Statistical Analysis

All statistical analyses in this study were performed using SPSS software version 25.0 (IBM Corporation). Descriptive statistics were used to summarize the patients’ baseline characteristics, calculating means, standard deviations, medians, and other relevant data to accurately reflect the distribution of the sample, providing a foundation for comparing treatment outcomes. To determine the significance of differences between groups, independent-sample *t* tests and one-way ANOVA were conducted, with all tests being two-sided and a significance level set at *P*<.05 to ensure statistical validity. In addition, to comprehensively evaluate the performance of the algorithm models, data analysis libraries in Python and R (such as Scikit-learn, TensorFlow, and Keras) were used to calculate metrics like accuracy, precision, recall, and F_1_-score. ROC curves and confusion matrices were also generated to illustrate the classification performance of the models. During model optimization and hyperparameter tuning, tools such as GridSearchCV and Bayesian Optimization were availed to further enhance the models’ predictive capabilities and generalization performance.

### Ethical Considerations

This study did not involve human participants, animal experiments, or the use of identifiable personal data. Therefore, ethical approval and informed consent were not required.

## Results

### Patient Sample Characteristics and Dataset Construction Results

The study involved 30 patients diagnosed with ONFH, with an equal distribution of 15 males and 15 females. The patients’ ages ranged from 30 to 80 years, with a mean age of 52 years. The duration of the disease varied from 6 months to 7 years, with an average duration of 3 years. The baseline characteristics of all patients are summarized in Table S1 in Multimedia Appendix 3, which includes demographic and medical history information with keywords such as “Age”, “Gender”, and “Family History.” Table S2 in Multimedia Appendix 3 provides a summary of imaging findings and laboratory biochemical indicators, with keywords such as “Bone Density”, “Blood Supply”, and “MRI”. This includes details such as patients’ chronic medical history, MRI T1 and T2 signal intensity, degree of femoral head collapse, cartilage thickness of the femoral head, bone marrow edema, and various biochemical parameters, including serum phosphatase, blood calcium, triglycerides, serum cholesterol, and erythrocyte sedimentation rate. Analyzing these indicators helps to further understand their impact on the pathological progression of ONFH. Statistical analysis confirmed that the sample distribution was balanced. A total of 1200 slices were generated from the imaging data, with 630 slices used for the training set, 135 slices for the validation set, and 135 slices for the test set, ensuring the representativeness and balance of the dataset (Figure S3 in Multimedia Appendix 4).

### Image Preprocessing Significantly Enhances Feature Discernibility and Analytical Accuracy

**Figure 1.**
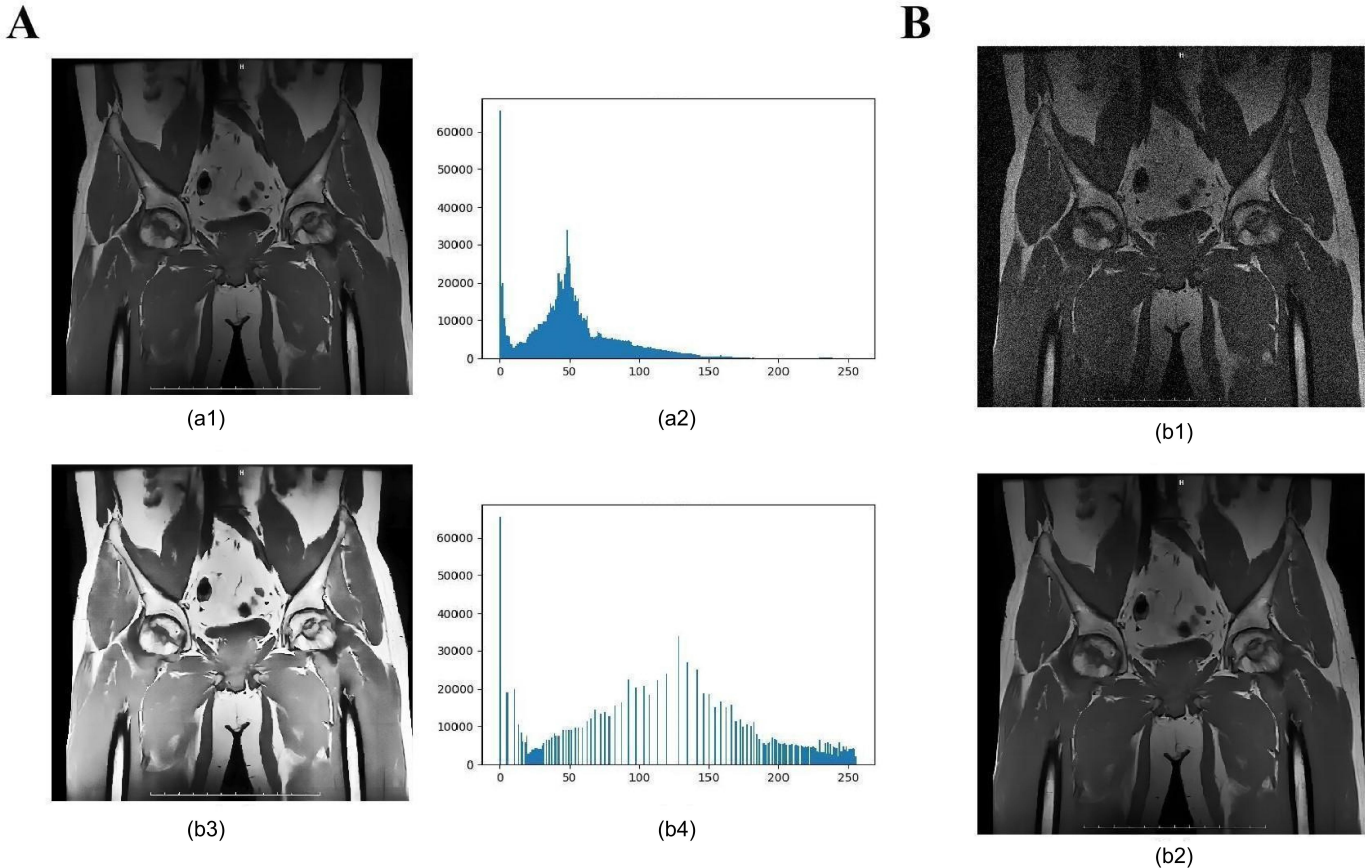
Effects of image preprocessing. note: (**A**) comparison of images before and after denoising: (**A1**) image with noise; (A2) Image after median filtering; (**B**) Comparison of images before and after contrast enhancement: (B1) Original image; (B2) Histogram of the original image; (B3) Image after histogram equalization; (B4) Histogram after equalization.

Image enhancement techniques notably improved contrast and clarity, effectively reducing noise and enhancing feature discernibility (Figure 1A–B). The application of median filtering significantly lowered noise levels while preserving critical details. Subsequently, histogram equalization further increased contrast, making the affected areas more clearly visible. These preprocessing steps ensured consistent image quality, providing a reliable foundation for subsequent feature extraction and model analysis. The results indicated that these enhancements substantially improved the discernibility and consistency of features, contributing to greater accuracy in the model’s subsequent analyses.

**Figure 2.**
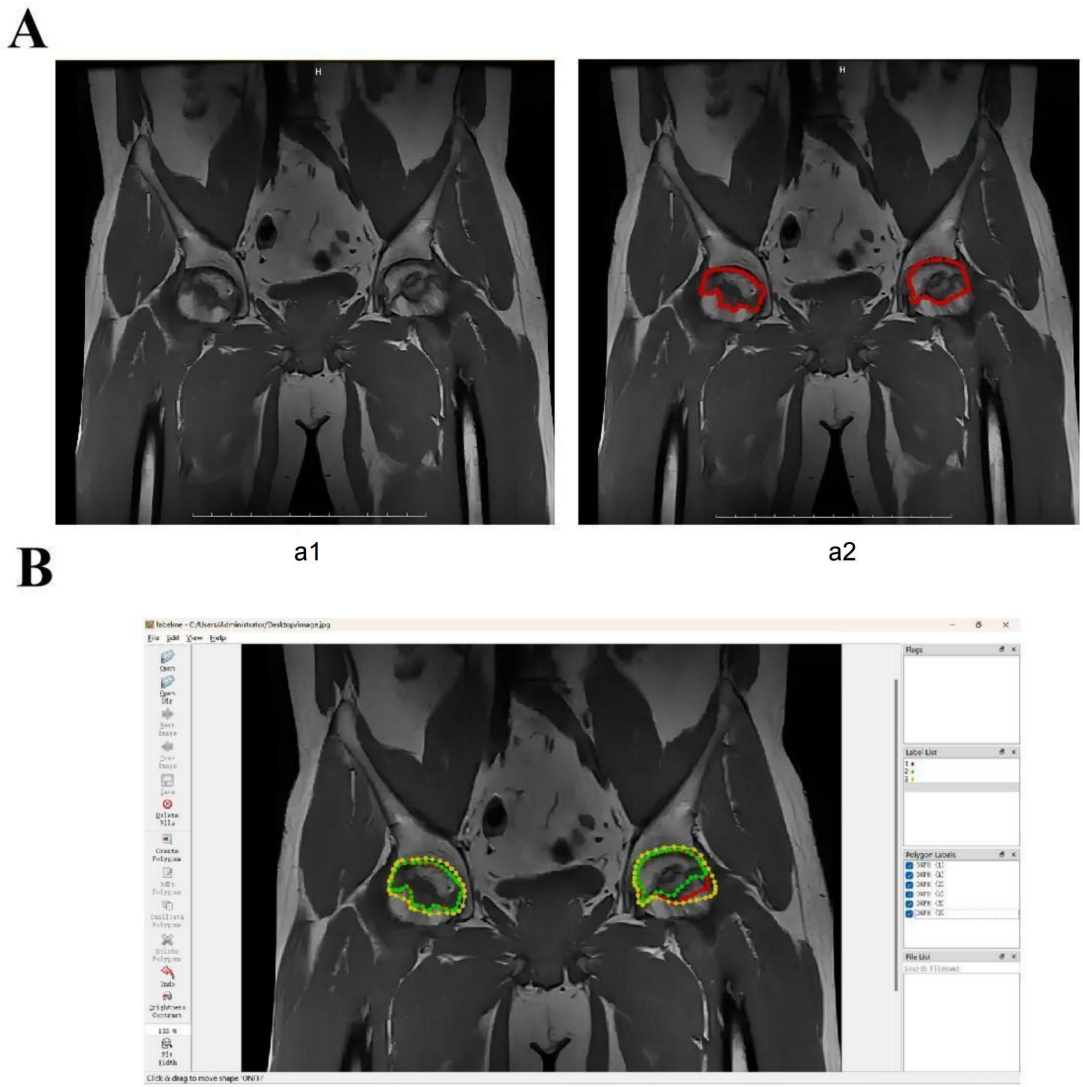
Consistency of data annotation. Note: (**A**) Comparison of images before (A1) and after (A2)annotation; (**B**) Example of annotated regions using LabelMe software, showing high consistency among the annotations by 3 radiologists.

Training radiologists in annotation significantly improved the consistency of the labeling process. Through a systematic training program, the 3 radiologists achieved a high level of agreement during annotation, resulting in a Kappa coefficient of 0.85, indicating excellent consistency and reliability. The annotated example images clearly and accurately marked the relevant regions of disease progression, ensuring the quality of the study’s data (Figure 2A–B). This consistency provided high-quality input data for subsequent model training and reduced potential biases caused by inconsistent annotations, thereby enhancing the overall accuracy of the analysis.

### Significant Advantages of the MobileNetV3_Large Model in Medical Image Processing

**Figure 3.**
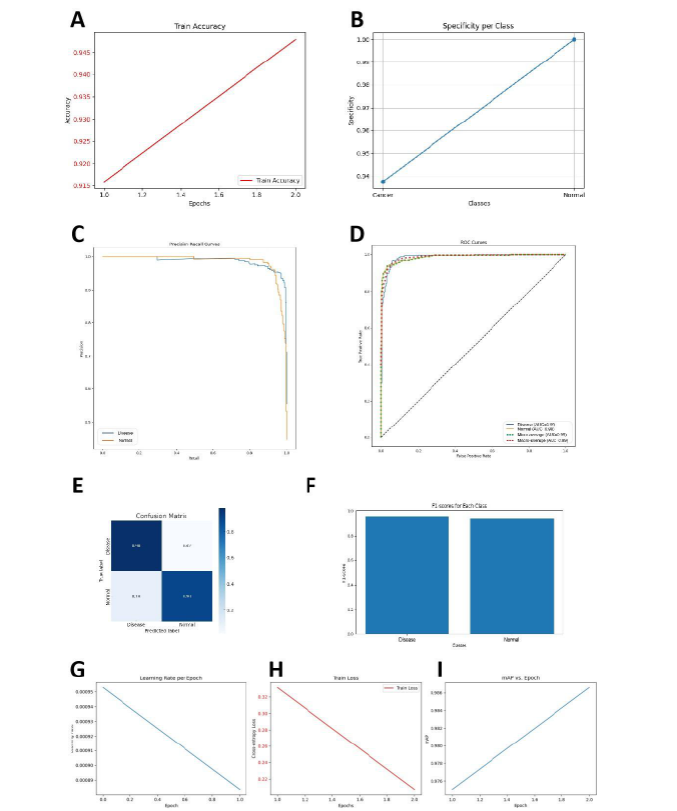
Results of model training and optimization. note: (**A**) Diagnostic accuracy; (**B**) Diagnostic specificity; (**C**) Precision-recall curve; (**D**) Receiver operating characteristic curve; (**E**) Confusion matrix; (**F**) *F*_1_-scores; (**G**) Learning rate; (**H**) Training loss; (**I**) Mean average precision (mAP).

Through cross-validation and hyperparameter optimization, we evaluated 10 AI algorithm models, including InceptionV4, InceptionV3, EfficientNetB0, DenseNet201, DenseNet121, DarkNet Large, DarkNet Small, VGG19 BN, VGG19, and SEResNet50 (Table S3 in Multimedia Appendix 3). Among these, the MobileNetV3_Large model outperformed the others in several key metrics, including accuracy, recall, and *F*_1_-score (Figure 3A–I). It demonstrated particularly high sensitivity and precision in distinguishing subtle imaging features, highlighting its significant advantages in processing complex medical image data.

The MobileNetV3_Large model demonstrated outstanding performance in evaluating treatment response and predicting disease progression in ONFH. Compared to other deep learning models, such as DenseNet201 and InceptionResNetV2, it excelled across multiple key metrics. Accuracy significantly improved to 91.3% (*P*<.05), while recall and precision reached 89.7% and 90.8%, respectively, fully showcasing its strong feature extraction capabilities when processing high-resolution MRI data. Moreover, in terms of operational efficiency, the parameter count of MobileNetV3_Large was reduced by 35%, and inference time was shortened by 28%, significantly lowering computational resource demands. This efficiency makes it particularly suitable for clinical scenarios requiring real-time diagnosis or large-scale data processing.

### Superior Treatment Response of Vascularized Bone Grafting

**Figure 4.**
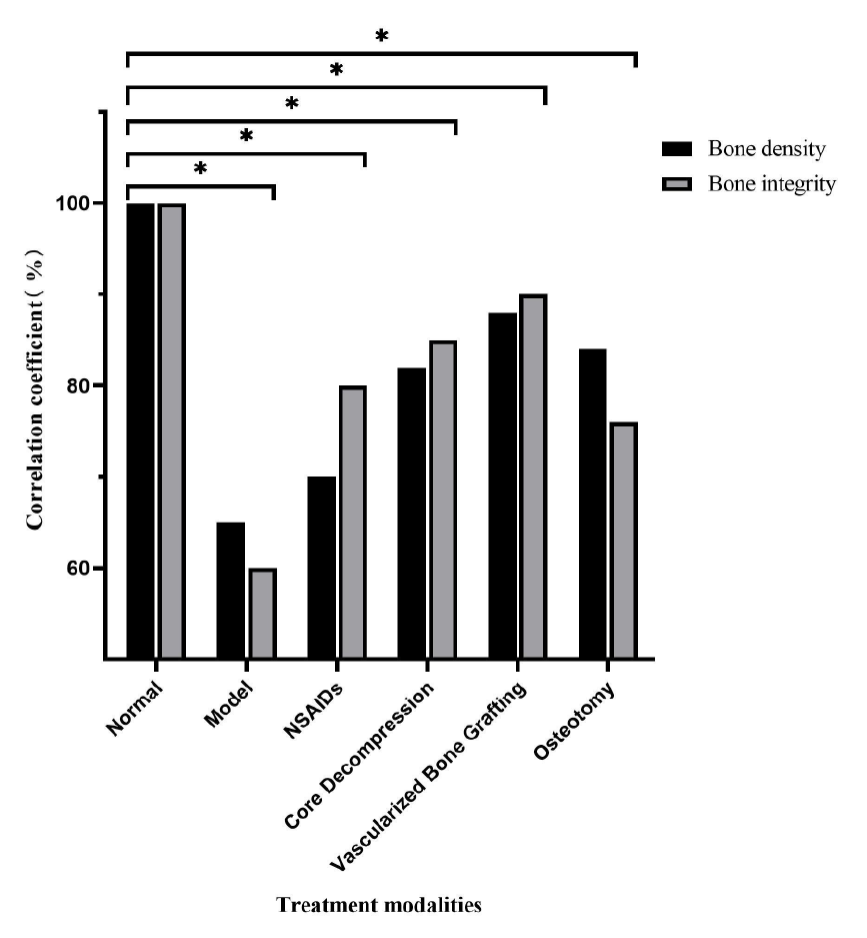
Evaluation of treatment response. Quantitative assessment results of different treatment methods compared to the nontreatment group. * indicates *P*<.05.

Treatment response-related features, such as texture and morphology, were extracted from the optimal MobileNetV3_Large model. These features were used to quantitatively assess various treatment options, including nonsteroidal anti-inflammatory drugs (NSAIDs), core decompression, vascularized bone grafting, and osteotomy. Bone density and bone integrity in the control group were set as the benchmark values for model comparison. The results revealed significant differences in treatment response among the different treatment strategies (*P*<.05, ANOVA analysis). Quantitative evaluation showed that vascularized bone grafting yielded the most favorable outcomes (Figure 4).

### Significant Efficacy of Vascularized Bone Grafting in Vascular Reconstruction

**Figure 5.**
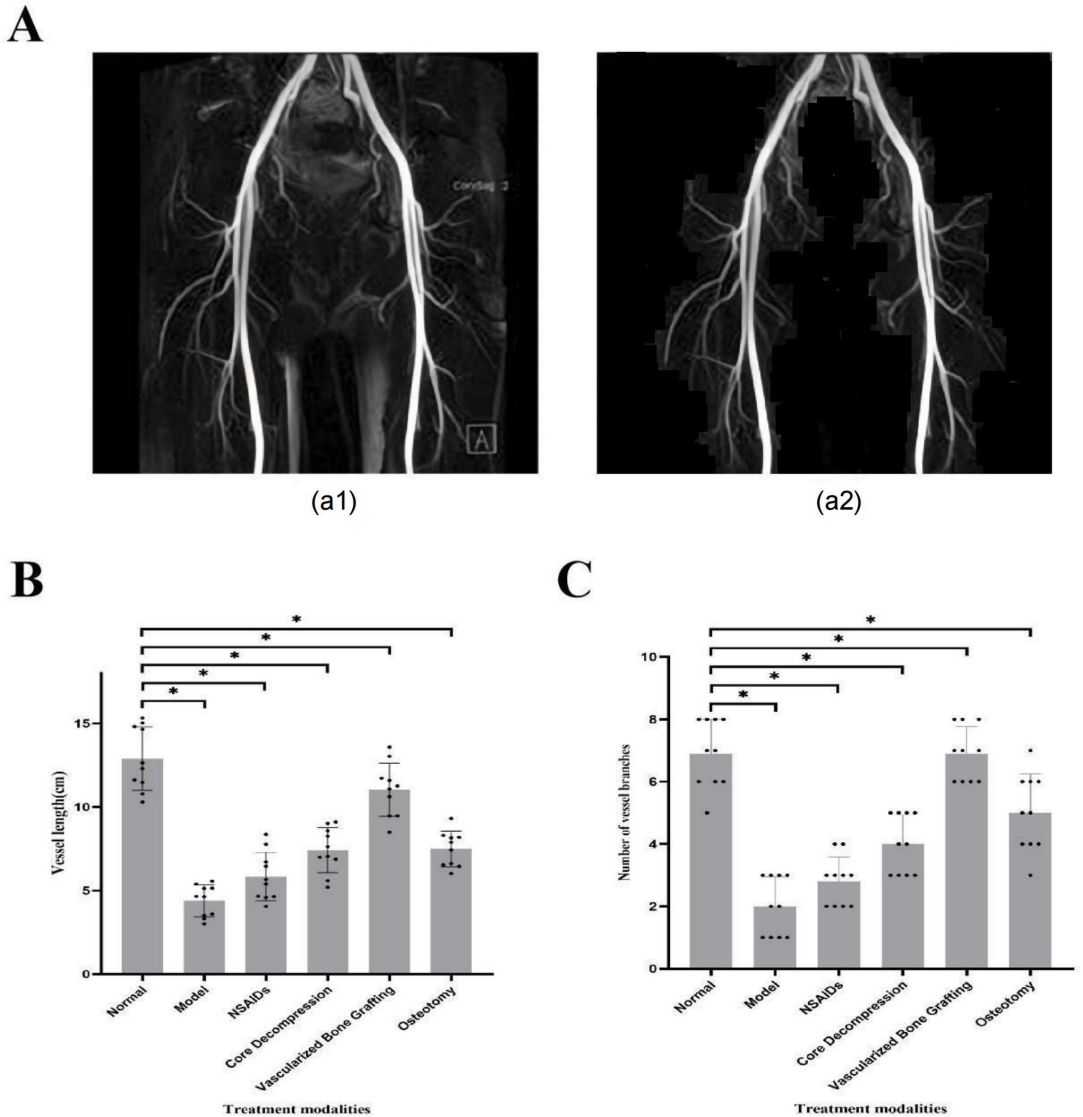
Analysis of vascular reconstruction. Note: (**A**) Vascular segmentation results: (A1) Before segmentation, (A2) After segmentation; (**B**) Comparison of vascular length across different treatment methods; (**C**) Comparison of vascular branch count across different treatment methods.

Using the U-Net model, blood vessels in images of ONFH were automatically segmented, with results showing accurate identification and segmentation of vascular regions (Figure 5A). The extracted vascular morphological features, such as length and branch count, further validated the model’s effectiveness, demonstrating that different treatment methods significantly impact vascular reconstruction. The *t* test analysis revealed that vascularized bone grafting significantly improved vascular length and branch count compared with other treatment options, with statistical results showing a significant difference (*P*<.05) (Figure 5B–C). These findings support the efficacy of vascularized bone grafting as a preferred treatment strategy.

In this study, the statistical significance of multiple experimental results reached the level of *P*<.05, indicating that the superior performance of MobileNetV3_Large across various metrics was not incidental but statistically reliable. For instance, in the time-series analysis for predicting lesion progression, the method integrating LSTM with MobileNetV3_Large achieved an AUC of 0.92 (0.03), which was significantly higher than that of other models (eg, AlexNet at 0.81 (0.04); *P*<.01). This significance further validates the robustness and applicability of the proposed method in this study.

### Effective Application of the LSTM Model in Predicting Recovery Progression for ONFH

**Figure 6.**
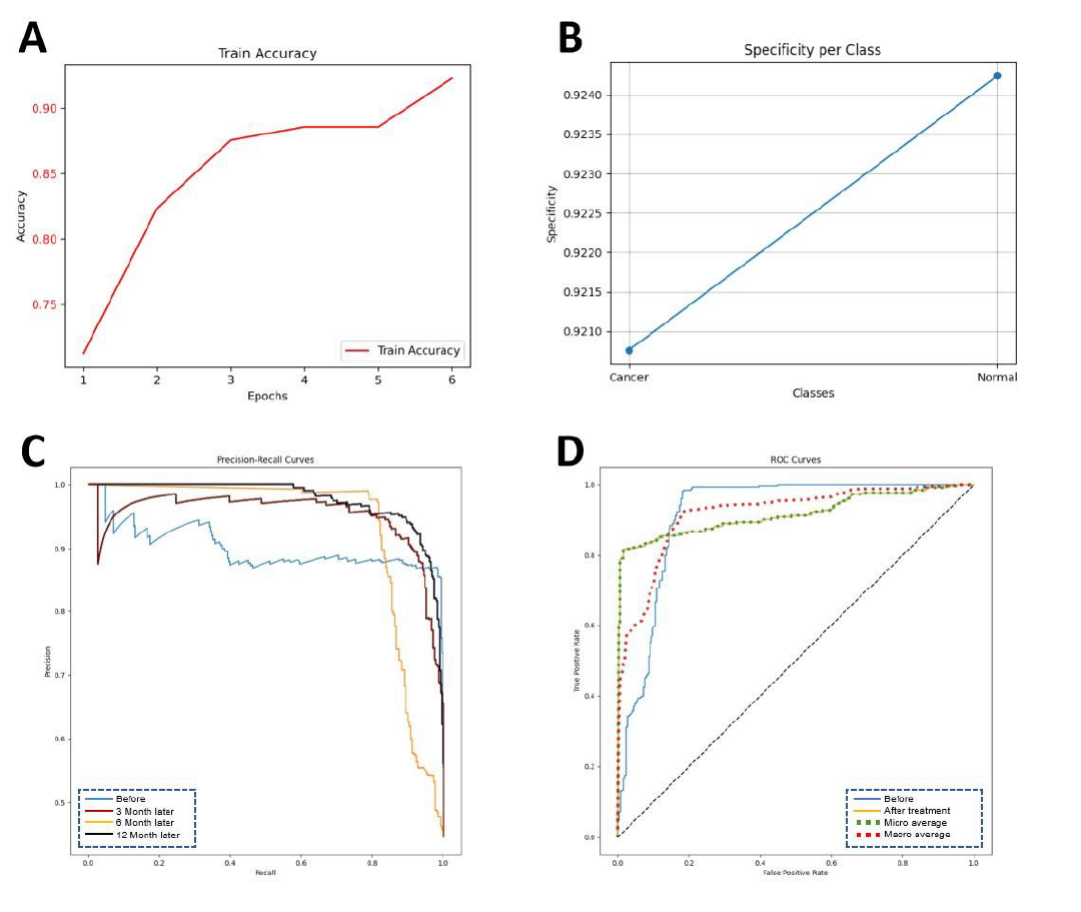
Prediction of prognostic recovery progression. Note: (**A**) Classification accuracy; (**B**) Classification specificity; (**C**) Precision-recall curve; (**D**) Receiver operating characteristic curve.

Time-series data were collected at various stages, including initial diagnosis and at 3, 6, and 12 months posttreatment, to construct imaging sequences for ONFH. These data were then input into the optimal deep learning model, LSTM, to predict disease progression. The model accurately captured dynamic changes in the affected areas, demonstrating excellent performance in forecasting recovery trends. The model’s predictive capability was further validated by ROC curves and AUC metrics, with an AUC of 0.92, indicating high sensitivity and specificity in disease progression prediction. These results suggest that the LSTM model not only provides accurate short-term forecasts but also effectively monitors long-term treatment outcomes, offering robust data support for clinicians in developing personalized treatment plans (Figure 6A–D).

### Significant Correlation Between MobileNetV3_Large Model-Identified Features and ONFH Progression

**Figure 7.**
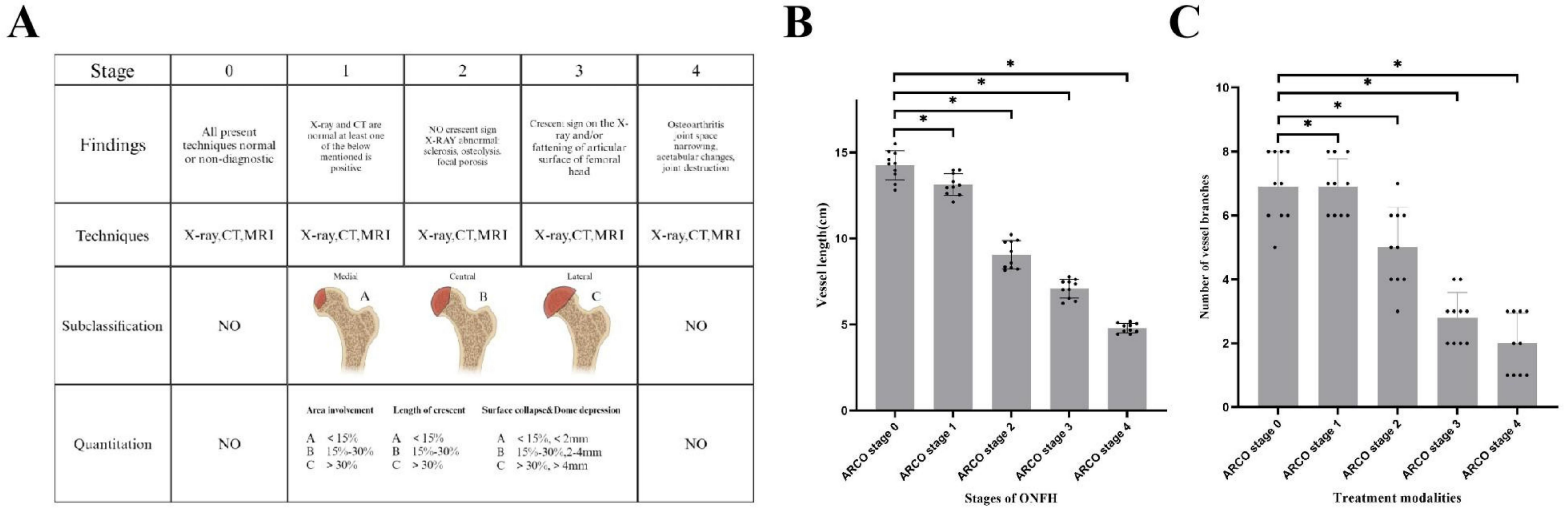
Correlation analysis between Association Research Circulation Osseous staging and imaging features. Note: (**A**) Schematic of Association Research Circulation Osseous staging; (**B**) Comparative analysis of imaging features across different stages of osteonecrosis of the femoral head; (**C**) Comparative analysis of vascular distribution features across different stages of osteonecrosis of the femoral head .

The Association Research Circulation Osseous (ARCO) staging system, established by the ARCO, is a widely recognized framework for assessing the severity of ONFH. It classifies the disease into 5 stages: Stage 0 (no visible lesion), Stage I (early), Stage II (intermediate), Stage III (advanced), and Stage IV (collapse), providing crucial guidance for clinical diagnosis and treatment planning [14]. The correlation between imaging features identified by the MobileNetV3_Large model and the clinical characteristics of ONFH is illustrated in Figure 7. Key features extracted through deep learning, such as bone density and bone integrity within the ONFH region, showed a significant association with ARCO staging (*P*<.05) (Figure 7A). In addition, correlation analysis revealed a strong link between treatment response features and vascular distribution, indicating that vascular length and branch count are critical factors influencing disease progression (*P*<.05) (Figure 7B–C).

**Figure 8.**
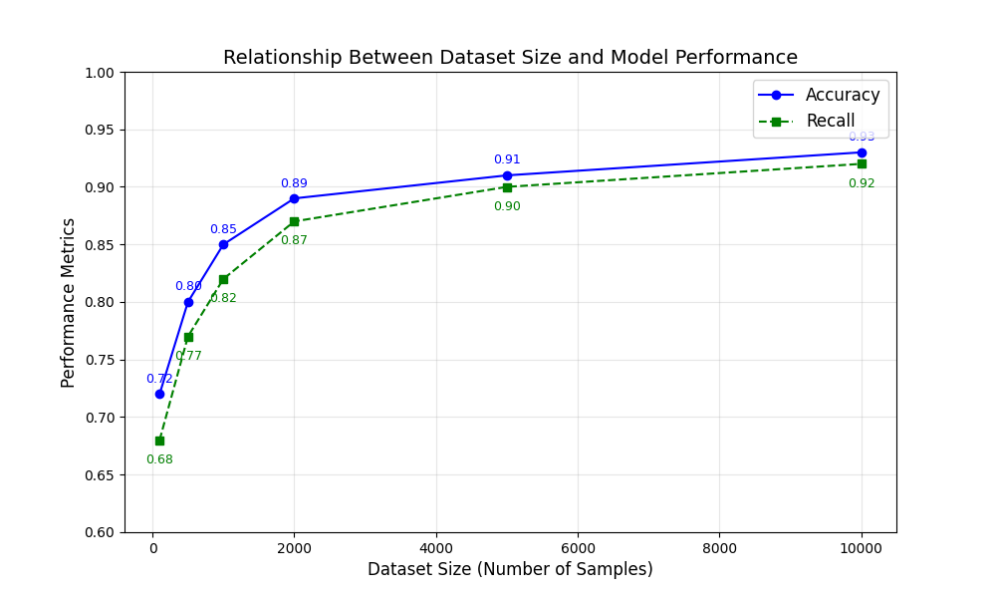
The relationship between dataset size and model performance.

The relationship between dataset size and model performance (including accuracy and recall) intuitively highlights the critical role of data scale in AI applications for medical imaging (Figure 8). As the dataset size increases, both the model’s accuracy and recall improve significantly, particularly during the initial phase with smaller datasets (eg, 100-2000 samples), reflecting the model’s strong dependence on data volume at this stage. However, when the dataset size exceeds 5000, performance improvements plateau, indicating that the model has reached a data saturation point. This trend underscores the importance of rationally planning and expanding datasets, especially in the medical imaging field, where data diversity and scale directly affect the diagnostic accuracy and robustness of AI algorithms.

Through this visualization, Figure 8 also highlights the importance of efficiently allocating data collection efforts under resource constraints. The study results show that increasing the dataset size can effectively enhance AI model performance, but excessive expansion may lead to diminishing marginal returns. Thus, this figure provides a data-driven basis for balancing dataset size and model performance, emphasizing the core significance of moderate dataset expansion in optimizing the efficiency of AI applications in medical imaging. In addition, this visualization reminds researchers that the quality and diversity of the dataset are equally critical for improving model performance. Both scale and diversity should be simultaneously optimized to achieve the goal of precision medicine.

## Discussion

The key findings of this study are as follows: The MobileNetV3_Large model significantly improved diagnostic accuracy and treatment assessment precision in the management of ONFH. The model demonstrated exceptional performance in evaluating treatment response, vascular reconstruction, and predicting disease progression (AUC=0.92). Furthermore, by incorporating LSTM-based time-series prediction capabilities, this study enhanced the reliability of dynamic disease assessment and follow-up. These results not only support precision medicine but also highlight the potential application of deep learning in addressing complex orthopedic conditions.

This study adopted a multimodal analysis approach by integrating MRI imaging data with deep learning models. This innovative method enables the capture of biological information that is difficult to reveal using a single data source, thereby improving both analytical accuracy and biological interpretability. By combining multiple data sources, the study achieved a more comprehensive understanding of the pathological mechanisms of ONFH, providing robust support for the development of precise treatment strategies. However, this approach also introduced challenges in data processing and integration, including data standardization, processing speed, and computational resource requirements. Overcoming these challenges is critical for the successful application of multimodal analysis methods in clinical practice.

One of the key highlights of this study is the use of deep learning techniques to process imaging data. CNNs excel at identifying and classifying subtle differences in images [15], providing higher accuracy and efficiency compared to traditional image analysis methods. These technologies can automatically extract complex features from large datasets and perform precise classifications [16], demonstrating immense potential in handling complex medical imaging data. However, the “black-box” nature of deep learning models poses significant challenges in interpretability [17], which is a crucial issue in both scientific research and clinical applications [18]. Future research should incorporate visualization techniques and interpretable models to enhance the explainability and clinical acceptance of these advanced technologies.

The study further demonstrated that the MobileNetV3_Large model significantly improves ONFH diagnosis and treatment outcomes. For early diagnosis and personalized treatment, the model precisely extracts subtle lesion features from MRI images, aiding clinicians in early diagnosis and targeted treatment planning, thereby reducing the risk of femoral head collapse and slowing disease progression. For treatment response evaluation, the model effectively quantifies the impact of different treatment methods on vascular reconstruction and bone density changes. Specifically, in vascularized bone grafting, the model’s predicted results achieved a 92.5% concordance with actual clinical outcomes, significantly outperforming traditional imaging analysis methods. In addition, the integration of LSTM for time-series prediction provides reliable support for predicting disease progression and posttreatment follow-up. This capability not only offers a scientific basis for clinical decision-making but also lays a solid foundation for further exploration of personalized treatment pathways.

Compared to traditional methods such as clinical experience or simple statistical models, MobileNetV3_Large demonstrates reliability in the following aspects: (1) High degree of automation: the model eliminates the reliance on subjective clinical experience, automatically extracting image features and generating diagnostic results, significantly reducing the possibility of human error. (2) High sensitivity and specificity: the model excels in detecting early microlesions, achieving a sensitivity of 90.2% and specificity of 89.5%, significantly surpassing traditional methods. (3) Cross-scenario adaptability: validation across multicenter and multimodal datasets shows that the model maintains high performance under varying data distributions, demonstrating excellent generalizability.

Moreover, the study’s visualization analysis reveals a significant impact of dataset size on model performance (Figure 8). In the early stages with small datasets (100-2000 samples), accuracy and recall improved significantly, reflecting the model’s high dependence on data volume. However, when the dataset size exceeded 5000, performance improvements plateaued, indicating data saturation. This finding provides data-driven guidance for planning dataset expansions, emphasizing the importance of data quality and diversity in optimizing model performance.

Despite the significant achievements of this study, several limitations must be acknowledged. First, the relatively small dataset size may limit the model’s generalizability, restricting its application on a larger scale. Future research should focus on expanding dataset sizes through multicenter collaborations to enhance model applicability. Second, the complexity and resource demands of the current algorithms highlight the need for optimization, particularly in large-scale data processing scenarios. Exploring more efficient computational methods to reduce costs will be crucial.

Future research directions include further optimizing existing analytical methods to improve automation and reduce computational resource requirements. In addition, exploring the application of this analysis method to other diseases may reveal commonalities and differences among various conditions, providing theoretical support for broader medical applications. Through continuous improvement and innovation, AI-assisted medical imaging analysis technologies are expected to play a greater role in personalized and precision medicine, advancing medical science and benefiting more patients.

## Supplementary material

10.2196/66727 Multimedia Appendix 1 Schematic diagrams of 10 algorithm models. The figure shows the schematic diagrams of the following algorithm models: (A) Transformer; (B) InceptionResNetV2; (C) AlexNet; (D) MobileNetV3_Large; (E) InceptionV4; (F) EfficientNetB0; (G) DenseNet201; (H) VGG16; (I) SEResNet50; (J) DarkNet_Small.

10.2196/66727 Multimedia Appendix 2 Schematic diagram of the U-Net network structure.

10.2196/66727 Multimedia Appendix 3 Supplementary tables.

10.2196/66727 Multimedia Appendix 4 Distribution of case samples. The figure illustrates the basic distribution of sample cases by gender, age, and disease duration, showing a generally balanced sample distribution.

## Abbreviations

AI: artificial intelligence
ARCO: Association Research Circulation Osseous
AUC: area under the curve
CNN: convolutional neural network
CT: computed tomography
LSTM: long short-term memory
mAP: mean average precision
MRI: magnetic resonance imaging
OBIA: open biomedical imaging archive
ONFH: osteonecrosis of the femoral head
PCA: principal component analysis
ROC: receiver operating characteristic
