# Supplementary figures and images for: Predictive Modeling of Osteonecrosis of the Femoral Head Progression Using MobileNetV3_Large and Long Short-Term Memory Network: Novel Approach
Source: JMIR Med Inform. 2025 Aug 6;13:e66727. doi: 10.2196/66727 (PMC12327698; doi:10.2196/66727)

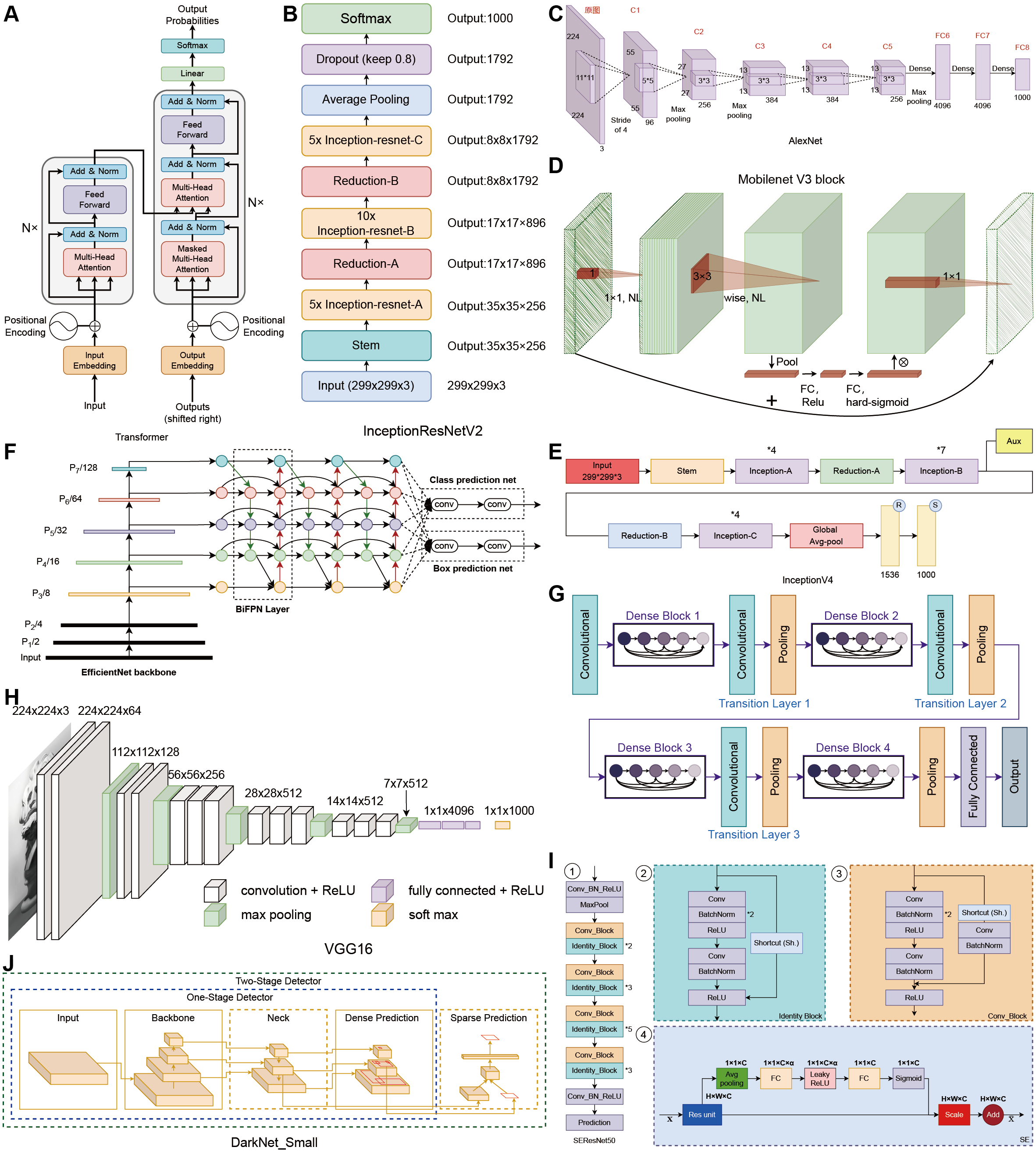

Supplement: Multimedia Appendix 1 [file medinform-v13-e66727-s001.png]

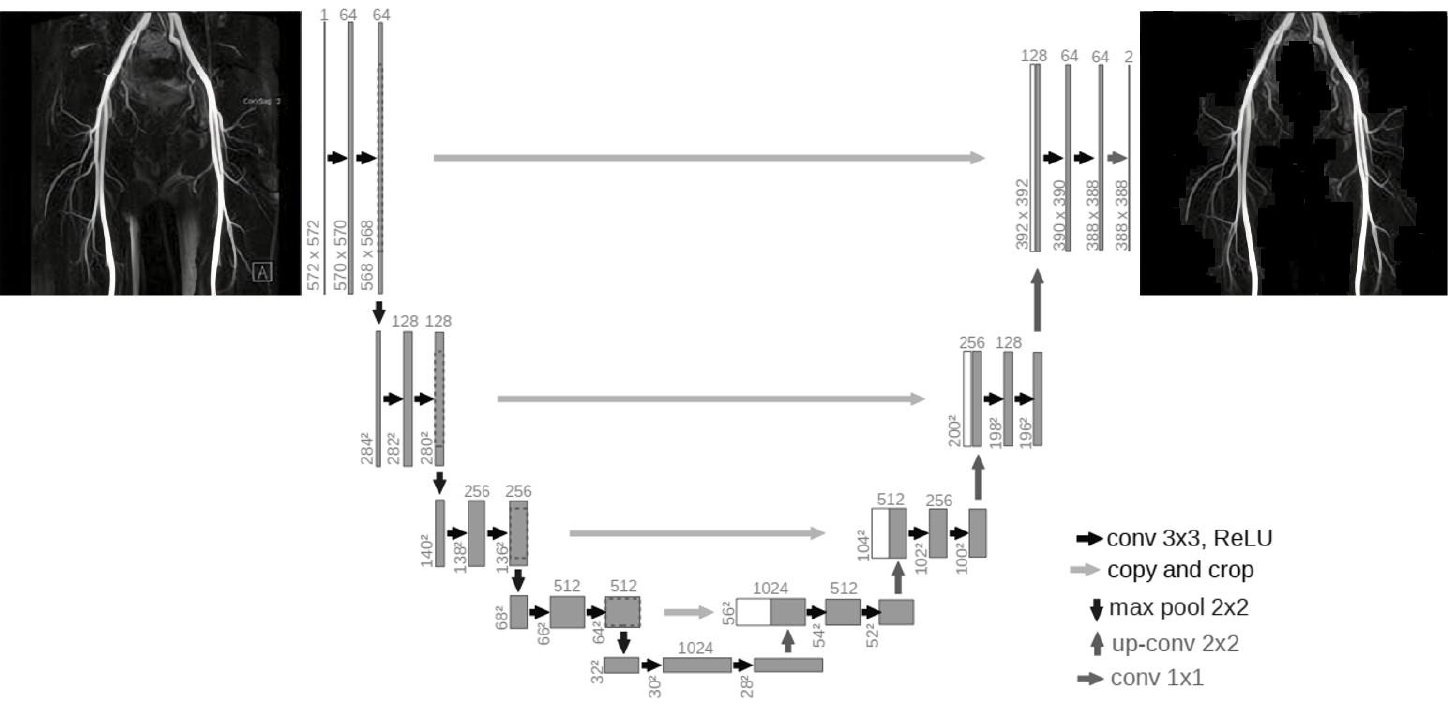

Supplement: Multimedia Appendix 2 [file medinform-v13-e66727-s002.png]

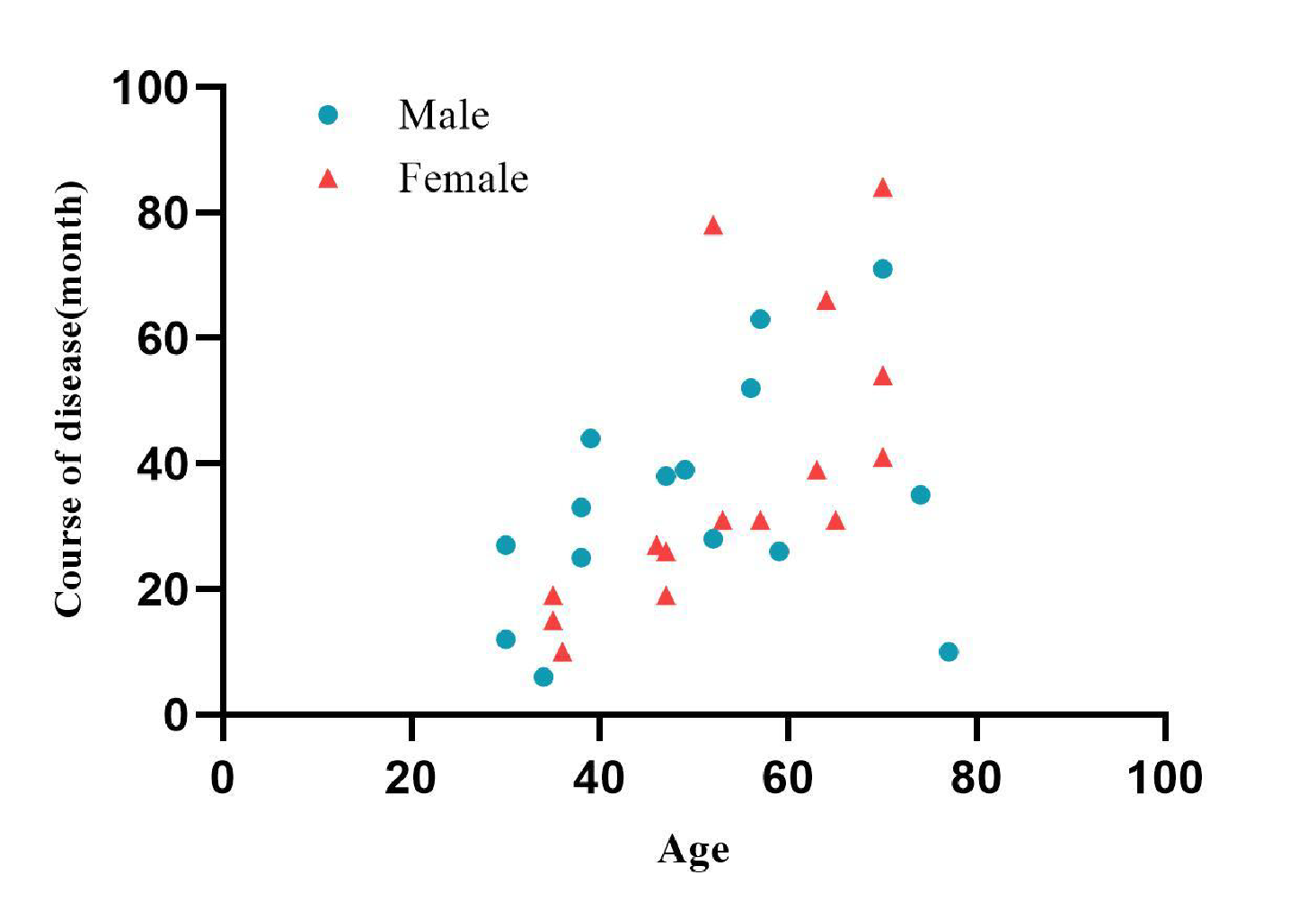

Supplement: Multimedia Appendix 4 [file medinform-v13-e66727-s004.png]
