# Supplementary material for: Predictive Modeling of Osteonecrosis of the Femoral Head Progression Using MobileNetV3_Large and Long Short-Term Memory Network: Novel Approach
Source: JMIR Med Inform. 2025 Aug 6;13:e66727. doi: 10.2196/66727 (PMC12327698; doi:10.2196/66727)
Supplement: Multimedia Appendix 3 [file medinform-v13-e66727-s003.docx]

**Table S1. Demographic and Medical History Characteristics of ONFH Patients.**

| Number | Age | Gender | Disease Duration (month) | Smoking History | Family History | History of Hypertension | History of Diabetes |
| --- | --- | --- | --- | --- | --- | --- | --- |
| 1 | 34 | Male | 15 | Yes | Yes | No | Yes |
| 2 | 77 | Male | 44 | Yes | Yes | Yes | No |
| 3 | 36 | Female | 27 | No | Yes | No | Yes |
| 4 | 30 | Male | 35 | No | Yes | Yes | No |
| 5 | 35 | Female | 19 | No | No | No | No |
| 6 | 35 | Female | 52 | Yes | No | Yes | No |
| 7 | 47 | Female | 12 | No | No | No | Yes |
| 8 | 38 | Male | 63 | Yes | Yes | Yes | No |
| 9 | 59 | Male | 66 | No | Yes | No | Yes |
| 10 | 47 | Female | 33 | Yes | No | Yes | Yes |
| 11 | 30 | Male | 28 | Yes | No | No | Yes |
| 12 | 46 | Female | 78 | No | No | No | Yes |
| 13 | 52 | Male | 41 | Yes | Yes | Yes | Yes |
| 14 | 57 | Female | 31 | Yes | No | Yes | Yes |
| 15 | 53 | Female | 6 | No | No | Yes | No |
| 16 | 65 | Female | 39 | Yes | Yes | Yes | No |
| 17 | 38 | Male | 71 | Yes | Yes | No | No |
| 18 | 74 | Male | 10 | Yes | Yes | No | No |
| 19 | 47 | Male | 25 | No | No | No | No |
| 20 | 49 | Male | 84 | Yes | Yes | Yes | No |
| 21 | 63 | Female | 38 | Yes | Yes | No | Yes |
| 22 | 70 | Female | 54 | No | No | Yes | Yes |
| 23 | 39 | Male | 26 | Yes | Yes | Yes | Yes |
| 24 | 56 | Male | 27 | No | No | No | Yes |
| 25 | 70 | Female | 39 | Yes | Yes | No | No |
| 26 | 57 | Male | 26 | No | Yes | No | Yes |
| 27 | 64 | Female | 31 | Yes | Yes | No | Yes |
| 28 | 70 | Male | 31 | Yes | Yes | Yes | No |
| 29 | 52 | Female | 19 | Yes | Yes | Yes | Yes |
| 30 | 70 | Female | 10 | Yes | No | No | Yes |

**Table S2. A summary of imaging findings and laboratory biochemical indicators**

| Number | Femoral Head Bone Density | Femoral Head Morphology | Femoral Head Blood Supply | MRI T1/T2 Signal Intensity | ARCO Stage | Bone Marrow Edema | Femoral Head Cartilage Thickness | Serum Phosphatase | Blood Calcium | Blood Triglycerides | Serum Cholesterol | Erythrocyte Sedimentation Rate |
| --- | --- | --- | --- | --- | --- | --- | --- | --- | --- | --- | --- | --- |
| 1 | Low | No Collapse | Good | Normal | 1 | No Edema | 3.021473555 | 28.36751398 | 9.342021057 | 1.357547706 | 4.447845 | 9.27825637 |
| 2 | Low | Moderate Collapse | Poor | Mixed Signal | 3 | Moderate Edema | 2.215285888 | 33.34821329 | 8.574935799 | 1.618283683 | 5.00632331 | 22.45568946 |
| 3 | Medium | Mild Collapse | Fair | Low Signal | 2 | Mild Edema | 2.779402198 | 25.40683016 | 9.41974856 | 1.230047497 | 5.264168398 | 11.53280417 |
| 4 | High | Moderate Collapse | Fair | Mixed Signal | 3 | Moderate Edema | 2.987485991 | 37.1582828 | 8.010096217 | 1.805885007 | 5.616405394 | 22.26288596 |
| 5 | Medium | No Collapse | Good | Normal | 1 | No Edema | 3.109472216 | 20.47632385 | 8.980612874 | 1.499244306 | 3.60622846 | 5.666910132 |
| 6 | Medium | Severe Collapse | Poor | High Signal | 4 | Severe Edema | 2.381550369 | 42.08934212 | 7.833616111 | 1.790886227 | 5.185805329 | 23.45440092 |
| 7 | Low | No Collapse | Good | Normal | 1 | No Edema | 3.684675289 | 26.46583343 | 10.48155694 | 1.14093747 | 3.690971277 | 6.941832214 |
| 8 | High | Severe Collapse | Poor | High Signal | 4 | Severe Edema | 2.153407966 | 42.511478 | 7.285749729 | 1.75253338 | 5.125573774 | 29.5853385 |
| 9 | High | Severe Collapse | Poor | High Signal | 4 | Severe Edema | 1.608983882 | 41.2194612 | 7.425545303 | 2.051710956 | 5.969026314 | 29.53939398 |
| 10 | Low | Moderate Collapse | Fair | Mixed Signal | 3 | Moderate Edema | 2.107577107 | 39.36268696 | 8.415273059 | 1.572775677 | 4.903664778 | 22.0806421 |
| 11 | Medium | Mild Collapse | Fair | Low Signal | 2 | Mild Edema | 3.345957554 | 34.31713481 | 8.889237182 | 1.490590466 | 4.15603151 | 11.64900468 |
| 12 | Low | Severe Collapse | Poor | High Signal | 4 | Severe Edema | 2.440185459 | 36.64008988 | 8.815363697 | 1.935065962 | 5.339048496 | 29.93095518 |
| 13 | High | Moderate Collapse | Poor | Mixed Signal | 3 | Moderate Edema | 2.136581658 | 31.7502251 | 9.225981972 | 1.706323367 | 5.497166425 | 15.75300636 |
| 14 | Medium | Mild Collapse | Fair | Low Signal | 2 | Mild Edema | 3.047048725 | 32.6691462 | 9.749431472 | 1.296731584 | 4.748771204 | 17.47871924 |
| 15 | Low | No Collapse | Good | Normal | 1 | No Edema | 3.523152102 | 27.30482857 | 9.070411122 | 1.111547458 | 3.602032665 | 11.74924695 |
| 16 | High | Moderate Collapse | Fair | Mixed Signal | 3 | Moderate Edema | 2.867311404 | 34.49458826 | 9.253026343 | 1.755376723 | 5.058606853 | 23.13481988 |
| 17 | High | Severe Collapse | Poor | High Signal | 4 | Severe Edema | 1.542617593 | 43.9002252 | 7.448054977 | 1.831555317 | 5.738923222 | 24.65031887 |
| 18 | High | No Collapse | Good | Normal | 1 | No Edema | 3.619035916 | 24.98780463 | 9.642224653 | 1.449192811 | 4.64098351 | 12.71119464 |
| 19 | Medium | No Collapse | Good | Normal | 1 | No Edema | 3.12360797 | 28.09242127 | 8.836344068 | 1.19864872 | 3.821924813 | 9.553409907 |
| 20 | Low | Severe Collapse | Poor | High Signal | 4 | Severe Edema | 2.152446813 | 43.20052559 | 8.275848358 | 1.736577588 | 6.423382537 | 20.93721272 |
| 21 | Medium | Moderate Collapse | Fair | Mixed Signal | 3 | Moderate Edema | 2.822489026 | 34.24001507 | 8.572986253 | 1.954767621 | 5.938270818 | 21.65966218 |
| 22 | Low | Severe Collapse | Poor | High Signal | 4 | Severe Edema | 1.741657219 | 36.94827164 | 7.579645708 | 2.077690068 | 5.27689913 | 28.11711092 |
| 23 | Medium | Mild Collapse | Good | Low Signal | 2 | Mild Edema | 2.720319095 | 30.75548864 | 8.359453697 | 1.242968591 | 4.103024782 | 18.1237681 |
| 24 | Low | Mild Collapse | Fair | Low Signal | 2 | Mild Edema | 2.755880565 | 33.23275098 | 9.347583253 | 1.281009608 | 4.21440293 | 13.61895902 |
| 25 | Medium | Moderate Collapse | Poor | Mixed Signal | 3 | Moderate Edema | 2.15328473 | 32.28114708 | 8.477009009 | 1.601649211 | 4.948359135 | 22.98145873 |
| 26 | Low | Mild Collapse | Good | Low Signal | 2 | Mild Edema | 3.009525593 | 33.98807766 | 9.276526271 | 1.264128282 | 5.246813634 | 13.72547054 |
| 27 | Medium | Mild Collapse | Fair | Low Signal | 2 | Mild Edema | 3.258346151 | 25.22490059 | 8.308394996 | 1.267800515 | 4.243745936 | 18.53715189 |
| 28 | Medium | Mild Collapse | Fair | Low Signal | 2 | Mild Edema | 2.90770035 | 28.18202851 | 8.982880528 | 1.370796229 | 4.084610836 | 11.22104835 |
| 29 | High | No Collapse | Good | Normal | 1 | No Edema | 3.619170572 | 28.3320039 | 9.898668209 | 1.253061506 | 3.60097276 | 12.60210705 |
| 30 | Low | No Collapse | Good | Normal | 1 | No Edema | 3.562757981 | 26.35980513 | 9.912741155 | 1.020585884 | 4.862203298 | 10.53862118 |

**Table S3. 10 Comparison of Features Across 10 AI Algorithm Models.**

| **Model Name** | **Data Preprocessing Performance** | **Adaptability** | **Overall Performance** | **Model Application Scope** |
| --- | --- | --- | --- | --- |
| Transformer | Excellent | High | High | Image Classification, Object Detection |
| InceptionResNetV2 | Good | High | High | Image Classification, Object Detection |
| AlexNet | Average | Low | Low | Image Classification, Object Detection, Image Segmentation |
| DenseNet201 | Good | Medium | Medium | Image Classification, Image Segmentation |
| InceptionV4 | Good | Medium | Medium | Image Classification, Image Segmentation |
| MobileNetV3_Large | Excellent | High | Highest | Object Detection |
| DarkNet Small | Average | Low | Low | Object Detection |
| EfficientNetB0 | Good | High | High | Image Classification, Object Detection |
| VGG16 | Good | High | High | Image Classification, Object Detection |
| SEResNet50 | Excellent | High | High | Image Classification, Image Segmentation |
